# Regulatory T cell frequency in peripheral blood of women with advanced cervical Cancer including women living with HIV

**DOI:** 10.1186/s12885-023-11345-9

**Published:** 2023-09-06

**Authors:** Devamani Chetty-Sebastian, Alain G. Assounga

**Affiliations:** 1 Clinical Medicine laboratory, Nelson R. Mandela School of Medicine, University of KwaZulu-Natal, 719 Umbilo Road, Durban, 4001 South Africa; 2 Dept of Nephrology, Div. of Internal Medicine, Nelson R. Mandela School of Medicine, University of KwaZulu-Natal, 719 Umbilo Road, Durban, 4001 South Africa

**Keywords:** Regulatory T cells, Cervical cancer, HIV, Human papillomavirus, Immunosuppression

## Abstract

**Background:**

Persistent high-risk Human papillomavirus (HR-HPV) infections are the main cause of cervical cancer. Cumulative evidence implicates regulatory T cells (Tregs) as a critical factor in the failure to eliminate HPV-induced cancers leading to their persistence and progression to cancer. Also, the WHO recognised cervical cancer as 100% attributable to persistent HR-HPV infection. The province of KwaZulu-Natal (KZN) in South Africa has a high prevalence of cervical cancer and HIV infection.

**Materials and methods:**

We evaluated Treg frequency in dual infection of HR HPV and HIV coinfection using phenotypic markers, CD4, CD25 and intracellular Foxp3, in the peripheral blood of 51 cervical cancer and 46 non-cervical cancer participants and evaluated the effect of HIV on regulatory T cell proportion. Peripheral blood mononuclear cells were surface stained with a cocktail fluorescent labelled CD4 and CD25 and subsequently with APC anti-human FoxP3 (eBioscience). Flow cytometry was performed with FACS analysis. Statistical analysis of results was done using Instat 3 program (GraphpadR). Tregs results were expressed as median ± interquartile range (IQR). Associations of cervical cancer with demographic, clinical and laboratory variables were evaluated by univariate and multivariate logistic regression analysis using SPSS version 27 (IBM).

**Results:**

Tregs frequency was significantly higher in individuals with cervical cancer (11.00 ± 19.79%) compared to controls (1.71 ± 8.91%) (*p* < 0.0001). HIV infection was associated with an increase in Tregs frequency. In controls a significant difference in Tregs frequency was noted between women living with HIV (6.00 ± 10.57%, n = 9) and those without HIV (1.30 ± 6.10%, n = 37), *p* = 0.0023. In multivariate logistic regression, Tregs frequency was significantly associated with cervical cancer after controlling for age, smoking, weight loss, presence of STI, HIV and HPV genotype.

**Discussion/Conclusion:**

Higher Tregs frequency was significantly associated with cervical cancer highlighting the immunosuppressive role of Tregs in cervical cancer. Treg frequency was more strongly associated with cervical cancer than HIV infection. We provide baseline data for monitoring Treg frequencies in response to new preventive and therapeutic strategies in the management of cervical cancer.

## Introduction

Globally, epidemiological data indicate cervical cancer is the 4th most prevalent cancer in women with an estimation of 604,000 new cases and 342,000 deaths with 90%, occurring in low-and middle-income countries in 2020 [1]. High-risk human papillomavirus (HR-HPV) are the aetiologic agents of cervical cancer development [2]. Women living with HIV infection are 6-fold more susceptible to cervical cancer development than women living without HIV [3].

Immunosurveillance and immune escape, play a critical role in the prevention and control of cancer [4, 5]. Immune cells involved in this process include CD4 + helper T cells (Th cells) and CD8 + cytotoxic T cells (CTL), among others. Th and CTL cells infiltrate and establish an adaptive immune defense in the tumour environment to eliminate cancer cells [6], thereby modulating disease progression [7]. A subset of CD4 + T cells referred to as regulatory T cells with the phenotype CD4 + CD25 + Foxp3 + T cells (referred to herein as Tregs) develop from the adaptive arm of the immune response. Tregs defend the host against inflammation and autoimmunity, but counteract anti-tumour immune responses [6, 8–10].

Increased infiltration of Tregs into tumour tissues can suppress anti-tumour responses [5, 11–13]. Also, Tregs in tumour-draining lymph nodes are suppressive in their function [14–16]. They exert their immunosuppressive action by competing with activated effector T cells (T-Effs) for interleukin (IL-2), to facilitate cell growth and early activation of immune response [16–18]. This action is a critical mechanism whereby they regulate the availability of IL-2 and other cytokines to suppress T-Effs action, thereby curbing effective immune defense [19, 20].

Broadly, Tregs comprise two subsets: natural FoxP3 Tregs (nTregs, derived from the thymus) and induced Tregs (induced peripherally) characterised by the expression of CD25 on the cell surface and the forkhead/winged transcription factor, FoxP3, in the nucleus [21–23].

Notably, the prevalence of Tregs is amplified in women with persistent HR-HPV infections [24] concomitant with cervical cancer but not in early precursor cervical intraepithelial neoplasia (CIN) stage [25]. Various Treg subsets (e.g.FoxP3 + CD127lo CD4 + T cell) can lower immune activation in infections such as HIV-1 [26].

HPV-induced cancers are associated with a failure to mount a strong HPV- specific T helper and cytotoxic T lymphocyte (CTL) response. In HPV-induced cancers there is a lack of CD8 + T cells migrating into tumours and an influx of Tregs into tumours [5, 27, 28]. High Tregs frequency in invasive carcinomas of HPV-positive patients suggest that Tregs prepare a niche conducive for HPV to persist and progress into invasive disease. Importantly, Tregs have an adverse effect on anti-tumour immunotherapy [28].

Sub-Saharan Africa bears a disproportionate burden of cervical cancer and harbours 67% of the global HIV burden [29]. South African women living with HIV and persistent HPV infections, are at a higher risk of developing cervical cancer than their negative counterparts [30, 31]. In KwaZulu-Natal (KZN) a province of SA, and the epicentre of the on-going HIV pandemic, women continue to present with advanced stages of cervical cancer [32].

This experimental study evaluated the frequency of Tregs in peripheral blood of women with and without cervical cancer, living with or without HIV infection. These results may contribute to our understanding of the natural history of Tregs in cervical cancer patients in a setting of single or dual infections.

## Materials and methods

### Selection and sample processing of study participants

This cross-sectional descriptive study received institutional ethical approval from The University of KwaZulu-Natal Biomedical Research Ethics Committee (BREC) number H097/04. Fifty-one newly histopathologically and clinically diagnosed women with cervical cancer attending the Antenatal Clinics at a district, regional and tertiary hospital in eThekwini, KwaZulu-Natal, SA. The lesion was staged according to the Federation of Gynaecology & Obstetrics (FIGO) into stages II (n = 17) and III (n = 31) at the Department of Pathology of a large central tertiary referral hospital. Inclusion criteria included age (> 18 years) with cervical cancer and not on radiotherapy or chemotherapy nor with any other type of cancer or with active per vaginal bleeding or menstruating or pregnancy. All experiments were performed in accordance with relevant guidelines and regulations. All experimental protocols were approved by BREC, University of KwaZulu-Natal (UKZN).

Forty-six female healthcare workers aged over 18 years with normal PAP smears were recruited as controls (Table 1). Informed consent was obtained from all participants involved in the study. A questionnaire covering: age, marital status, contraceptive use, parity, smoking history and history of sexually transmissible disease was administered (Table 1). Since their HIV status was unknown, they were advised of pre and post-HIV counselling by the hospital’s HIV-trained staff.

### Procedure

Cervical biopsies (approximately 3 mm) were obtained from cervical cancer participants. Cervical biopsies were immediately snap-frozen in liquid nitrogen/dry ice, transported to the laboratory and stored at -80ºC until required for deoxyribonucleic acid (DNA) extraction used for detection and genotyping of HPV.

#### HPV DNA, extraction, detection and genotyping

DNA extraction from cervical swabs and cervical biopsies was done using the QIAamp DNA Micro Kit, and QIAamp DNA Mini Kit (QIAGEN, Inc., Valencia, CA), respectively, as per manufacturer’s instructions. DNA quantified using the NanoDrop^R^ ND-1000 spectrophotometer (ThermoFisher Scientific, USA). Detection and Genotyping of HPV DNA carried out using Linear Assay (Roche Molecular Systems, Inc., Branchburg, NJ) which identifies 37 HPV genotypes comprising high risk (HR) (16, 18, 26, 31 ,33, 35, 39, 45, 51, 52, 53, 56, 58, 59, 66, 67, 68, 69, 70, 73, 82, IS39 ), and low risk (LR) variants ( 6, 11, 40, 42, 54, 55, 61, 62, 64, 71, 72, 81, 83, 84, CP6108.

#### Staining for CD4 + CD25 + FoxP3 + T regulatory cells

Participants’ peripheral blood mononuclear cells (PBMCs) were obtained from blood samples by Ficoll Hypaque centrifugation performed at 1440 rpm using Eppendorf entrifuge model 5810 with a rotor of a radius of 17.3 cm. This corresponds to 400 g (g = rpm^2^ x radius x 1.118 × 10^− 5^ = 1440^2^ × 17.3 × 1.118 × 10^− 5^ = 400). The cells were stored at -80^o^C until being used. Participants’ PBMCs were then, thawed to determine the frequency of CD4 + CD25 + FoxP3 + T cells.

A Human Regulatory T cell Staining Kit (Cat No. 88-8998) was used for staining natural Tregs and included labelled monoclonal antibodies against CD4, CD25, and FoxP3. The reagents included, APC- labelled FoxP3 (Clone PCH 101), FITC- labelled CD4, PE-labelled CD25, in a phosphate buffer, ph 7.2, 150 Mm NaCl, 0.09% NaN_3,_ 0.2% BSA, Clone PCH101, Isotype: Rat IgG2a, (Cat.No.88-8998), (eBioscience, San Diego, CA,USA).

The test was performed as per the manufacturer’s instructions. By the use of the combination of these markers, the frequency of Treg cells was determined in PBMCs. Each test was performed using 3 tubes with the required number of 3 × 10^6^ PBMCs per test. Briefly, the procedure is as follows:

Tube 1-control − 1 × 10^6^ PBMCs, with PBS medium only.

Tube 2- control − 1 × 10^6^ PBMCs FITC- labelled anti-CD4, PE-labelled anti CD25 plus APC labelled rat IgG2a isotype control.

Tube 3-1 × 10^6^ PBMCs FITC- labelled CD4, PE-labelled CD25 plus APC labelled anti-human FoxP3 (PCH101).

To each labelled tube containing 100 µl of prepared cells (1 × 10^6^), 20 µl Via-Probe^™^ (Cat. No 555,816, BD Biosciences, San Jose, CA), a cell viability probe for dead cell exclusion was added to each tube. After the tubes were incubated at RTº for 10 min, a 20 µl cocktail of FITC anti-human CD4 (1ug-RPA-T4) and PE anti-human CD25 (0.125ug-BC96), was added to each tube and incubated for 30 min in the dark at 4ºC.

After incubation, cells were washed in cold flow cytometry staining buffer and centrifuged for 5 min at 300-400xg. After decanting, cell pellets were pulse vortexed, resuspended in the residual buffer, and 1ml Fixation/Permeabilisation buffer was added, followed by brief pulse vortexing. The tubes were incubated for 60 min in the dark at 4ºC, after which the tubes were washed twice in permeabilisation buffer, centrifuging and decanting of supernatant between the washes.

The pellets were resuspended in the residual buffer and mixed by pulse vortexing, followed by the addition of 20 µl anti-human FoxP3 (PCH101) antibody (tube 3) and 20 µl rat isotype control (tube 2) into the appropriate tubes. The tubes were incubated for 30 min in the dark at 4ºC, followed by two washes with permeabilisation buffer, then centrifugation. The supernatant was decanted, and the procedure was repeated. The pellets were then suspended in 500 µl of Flow Cytometry Staining Buffer and analysed on FACSCalibur (Beckton Dickinson, BD Biosciences, San Jose, CA).

#### Flow cytometry analysis

**Fig. 1:**
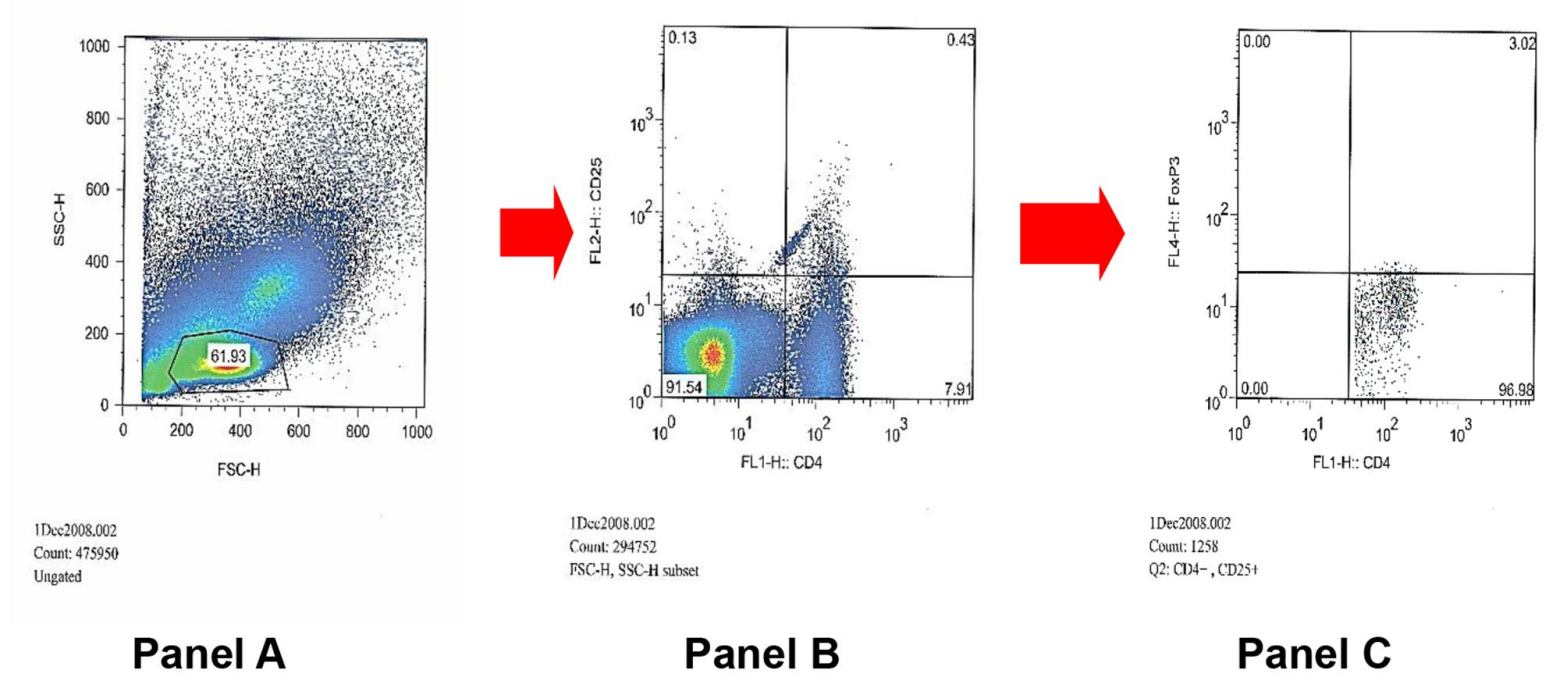
Dot Plot demonstrating 3.02% of non-specific binding (NSB) cells as a proportion of CD4 + CD25 + cells (Upper Right quadrant, Panel C) in a cervical cancer participant. **Panel A**: Dot plot representation of PBMC according to size (x-axis = forward scatter) and granularity (y-axis = side scatter); the gated region corresponds to lymphocytes. **Panel B**: Dot plot showing gated lymphocytes from panel A, stained with CD4 (x-axis) and CD25 (y-axis). **Panel C**: Dot plot showing gated double CD4 and CD25 positive cells (upper right quadrant in panel B) further stained with Isotype control (NSB/dead cells). The upper right quadrant represents cells (positive for CD4, CD25 and NSB)

**Fig. 2:**
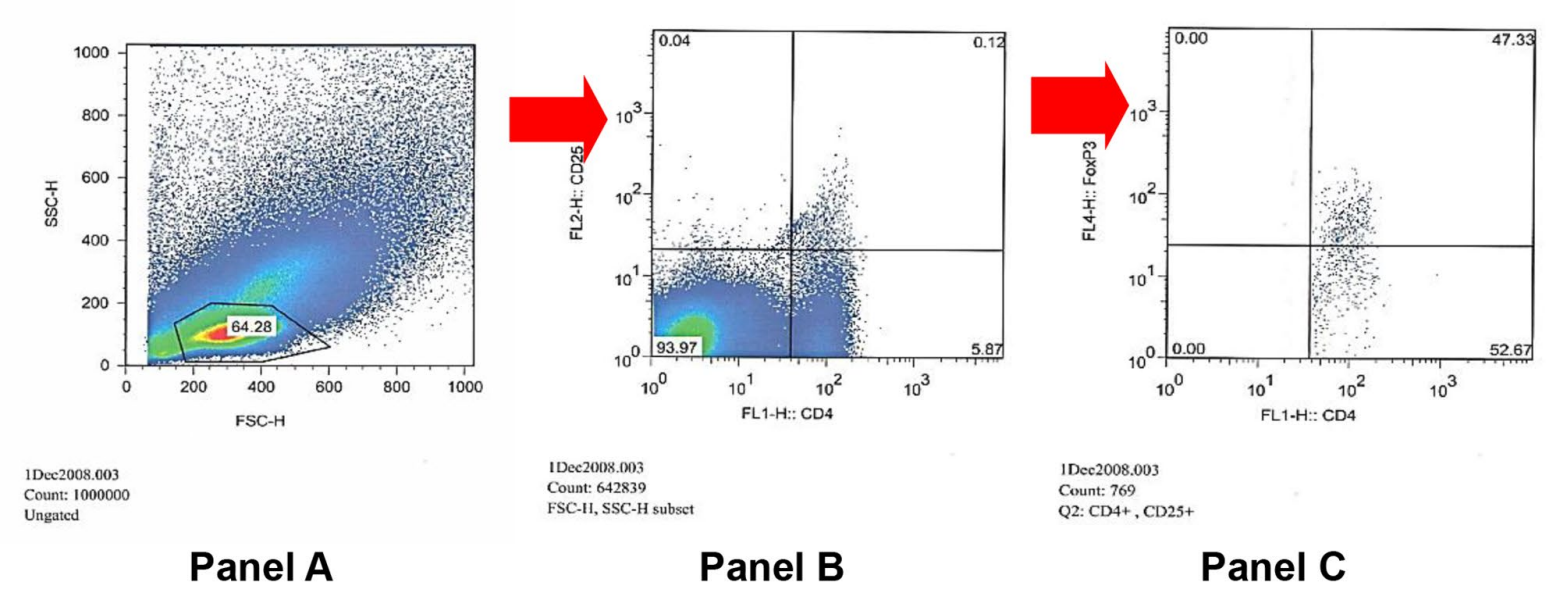
Dot Plot demonstrating FoxP3 in the same cervical cancer participant shown in Fig. 1. 47.33% positivity of FoxP3 as a proportion of CD4 + CD25 + cells (upper right quadrant, Panel C). **Panel A**: Dot plot representation of PBMC according to size (x-axis = forward scatter) and granularity (y-axis = side scatter); the gated region corresponds to lymphocytes. **Panel B**: Dot plot showing gated lymphocytes from panel A, stained with CD4 (x-axis) and CD25 (y-axis). **Panel C**: Dot plot showing gated double CD4 and CD25 positive cells (upper right quadrant in panel B) further stained with FoxP3. The upper right quadrant represents Treg cells (positive for CD4, CD25 and FoxP3).

Calibration beads (Becton Dickinson, San Jose, CA), were used for the calibration of the cytometer, FACSCalibur (Becton Dickinson, San Jose, CA). Owing to the fixation and permeabilisation procedures, the FSC/SSC distribution of the cell populations was different from that of live cells. Therefore, the gate and voltage had to be modified. Samples (10^6^ events) were acquired and subsequently analysed using FlowJo^™^ software (Flowjo, Oregon, CA) was used to analyse acquired data from FACS calibur FACS Calibur. The gating strategy is summarised in Figs. 1 and 2. The frequency readings from the specific antibodies tube FoxP3 (Fig. 2, Panel C, upper right) minus that from the non-specific binding tube (Fig. 1, Planel C, upper right) represents the final test analysis of live cells of each tested specimen.

#### Statistical analysis

Statistics were performed using Instat 3 (Graphpad^R^). The distribution of data in each group was first tested for normality. For group comparison, parametric tests were used when the distributions passed the normality test, while non-parametric tests were applied in cases where the normality test failed. Results were graphically presented using Graphprism^R^ (Graphpad^R^). Data were expressed as median ± interquartile range (IQR). Differences between the groups were then tested by a one-way t-test or non-parametric Mann-Whitney test; *p* < 0.05 was considered significant. Univariate and multivariate logistic regression analyses were conducted using IBM SPSS software to evaluate the association of variables studies with cervical cancer.

## Results

### Characteristics of participants is shown in Table 1

**Table 1:** Comparison of possible risk factors and clinical features between Cervical Cancer and Control Participants

Participants	Cervical cancer n = 51	Controls (Women without cervical cancer) n = 46	*p* values
**Age (median ± IQR%)** **Total (n)**	46 ± 20 51	37 ± 13 46	*p* = 0.0974
**Marital status: Married n (%)**	19 (38%)	22 (47.8%)	*p* = 0.4108
**Contraception**: **Yes n (%)**	23 (45.1%)	10 (21.7%)	*p* = 0.0182*
**Parity (median ± IQR%)** **Total (n)**	4 ± 3 51	2 ± 2 46	*p* < 0.001
**CD4 count (median ± IQR%)** **Total (n)**	231.5 ± 73.8 51	331.0 ± 275.0 46	*p* = 0.0272
**HIV Positive n (%)**	23 (46%)	9 (19.6%)	*p* = 0.0055*
**HPV genotypes: HR n (%)** **MR n (%)** **LR n (%)** **Neg n (%)**	20 (44.4%) 24 (53.3%) 1 (2.2%) 0 (0%)	3 (6.5% 10 (21.7%) 7 (15.2%) 26 (56.5%)	*p* < 0.0001*
**Smoking: Yes n (%)**	6 (12%)	10 (21%)	*p* = 0.2766
**STI: Yes n (%)**	12 (25%)	5 (12.2%)	*p* = 0.1069
**Loss of weight n (%)**	31 (67.4%)	2 (4.5%)	*p* < 0.0001*
**Tregs frequency** **(median ± I QR%)**			
**- Total** **- FIGO Stage II n = 17** **- FIGO Stage III n = 31**	11.00 ± 19.79% 9.31 ± 21.02% 12.12 ± 20.21%	1.71 ± 8.91%	*p* < 0.0001* *p* > 0.05 between stages II &III

*p* values < 0.05 are considered statistically significant; Test: Unpaired T-test or Chi-square test; * = significant

HR = high risk, LR = low risk. MR = mixed HR and LR; FIGO = Federation of International Gynaecology and Obstetrics

### Representative dot plot graphs of non-specific binding and frequency of tregs in participants

The CD4 CD25 positive population was analysed to determine the frequency of non-specific binding for FoxP3 (dead cells) using isotype control (upper right quadrant Fig. 1 = 3.02%) or total FoxP3 binding using anti-FoxP3 (upper right quadrant Fig. 2 = 47.33%).

Therefore, the live Treg frequency is 47.33% (Fox P3 frequency in Figs. 2) – 3.02% (Non-specific binding/ dead cells in Fig. 1) = 44.31%.

This was done for all participants in the study, and the resulting Tregs frequency was subsequently used in analysis thereafter.

### Frequency of CD4 + CD25 + FoxP3 + T cells (Tregs) in participants

Tregs frequency in peripheral blood of 51 cervical cancer and 46 control participants was evaluated using Foxp3 as a specific marker for Tregs. A significant increase in Treg frequency expressed as FoxP3 + Tcells as a proportion of CD4 + CD25 + cells was recorded in women with cervical cancer (11.00 ± 19.79%) compared to the controls (1.71 ± 8.91%), (*p* < 0.0001) (Table 1). There was no significant difference in Tregs frequency between cervical cancer stage II (n = 17) and stage III (n = 31) (9.31 ± 21.02% and 12.12 ± 20.21%; *p* > 0.05), respectively (Table 1).

### Frequency of Tregs in Human Papillomavirus infection

**Fig. 3:**
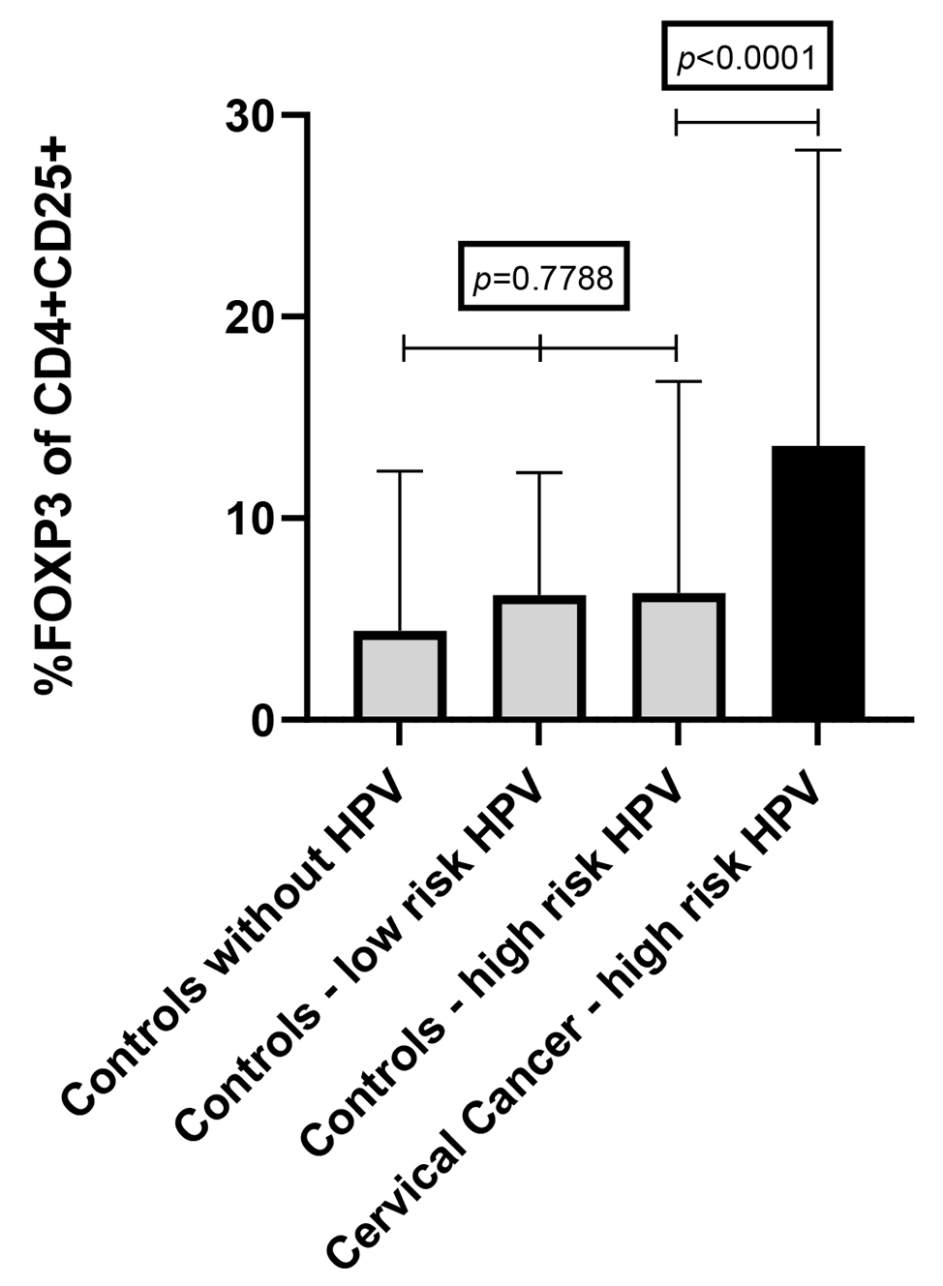
The bar graph demonstrates the frequency of T regulatory cells as a frequency of CD4 + CD25 + FoxP3 T cells according to HPV types in cervical cancer patients and controls women. No significant difference in Tregs frequency in controls for HPV infections (Kruskal-Wallis (p = 0.7788). Tregs frequency was significantly high in HR HPV cervical cancer compared to controls using the Mann-Whitney test, *p* < 0.0001). Bars represent the median, and error bars represent the interquartile range (IQR).

In controls, there was no significant difference in Tregs frequency between negative HPV women (0.73 ± 7.79%), and HPV low-risk (4.12 ± 11.34%) and HPV high-risk (1.72 ± 9.19%) types, (Kruskal Wallis, *p* = 0.7788). In women with cervical cancer, since all were infected with high-risk HPV, we could not perform this analysis. Overall, of all participants infected with HR HPV, Tregs frequency was significantly higher in cervical cancer (10.90 ± 15.97%) than in controls (1.72 ± 9.19%), *p* = 0.0001). Mixed Infections (MI) were included in the HR group (Fig. 3).

### Frequency of Tregs in HIV-positive participants

**Fig. 4:**
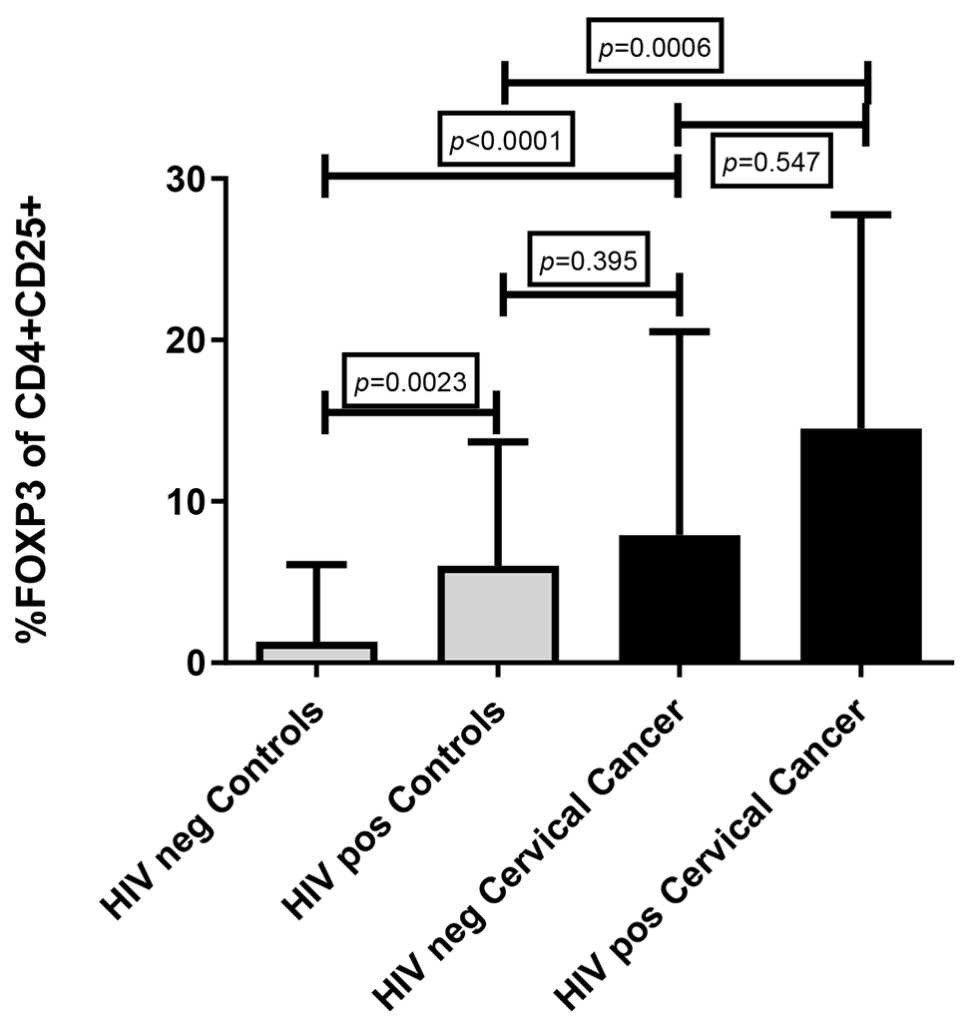
**T regulatory cell frequency in cervical cancer women according to HIV status**. The bar graph demonstrates a significant difference in Treg frequency between HIV (+) controls and HIV (+) cervical cancer, *p* = 0.0006. In controls, Treg values between HIV (+) and HIV (-) were significant *p* = 0.0023. However, Treg frequency was significant between HIV (-) cervical cancer and HIV (-) controls, *p* < 0.0001. No significant difference between HIV (+) cervical cancer and HIV (-) cervical cancer, *p* = 0.547and between HIV (+) cervical cancer and HIV (-) controls, *p* = 0.395. Non-parametric Mann-Whitney test. Bars represent the median, and error bars represent the interquartile range (IQR)

In 97 of the samples tested for Tregs frequency, 23 of the 51 women with cervical cancer were HIV positive, whilst 9 of the 46 controls were living with HIV. Regarding HIV, a higher Treg frequency was observed in women with cervical cancer compared to HIV-positive controls, *p* = 0.0006 (Fig. 4). In women with cervical cancer, Treg frequency between HIV infected (14.50 ± 23.64%, n = 23) and non-infected was not significant (7.91 ± 17.45%, n = 26), *p* = 0.547. In controls, a significant difference in Treg frequency was noted between the HIV-positive (6.00 ± 10.57%, n = 9) and HIV-negative (1.30 ± 6.10%, n = 37), *p* = 0.0023. Overall in all women living without HIV, Treg frequency was significantly higher in cervical cancer women (7.91 ± 17.45%, n = 26) compared to controls (1.30 ± 6.10%, n = 37), *p* < 0.0001. Overall, in women living with HIV, no difference in Treg frequency between women with cervical cancer (14.50 ± 23.64%, n = 23) and controls (6.00 ± 10.57%, n = 9), p = 0.395, (Fig. 4), was recorded.

### Frequency of tregs according to CD4 count

Participants were stratified into three groups, namely, < 200, 201–350 and > 350 cells/mm^3^. In the HIV-infected cervical cancer women, no statistically significant difference in Treg frequency according to CD4 count was recorded. The apparent drop of Tregs in patients with CD4 counts between 201 and 350 is, in fact, not significant as the p-value is p = 0.767(Kruskall-Wallis). The non-significance may be due to the low number of patients in this group (n = 8). Overall, in women living with HIV and having a CD4 count > 350, Treg frequency was significantly greater in cervical cancer than in controls, *p* = 0.0077 (Mann-Whitney) (Table 2). In view of the small number of participants in various groups, these results need to be confirmed in a large group. They are inconclusive.

**Table 2:** Frequency of CD4 + CD25 + Foxp3 + regulatory T cells (Tregs) in relation to CD4 count in HIV-infected Participants

CD4 count	Cervical Cancer n	Tregs (Median ± IQR%)	controls n	Tregs (Median ± IQR%)	*p* value
< 200	8	17.50 ± 19.01	1	-	
201–350	8	2.18 ± 10.12	1	-	
> 350	6	18.00 ± 26.17	6	5.60 ± 19.28	0.0077*
*p*-value		0.767**			

*Mann Whitney test, between cervical cancer patients and controls in the > 350 CD4 counts, *p* = 0.0077. **Kruskall-Wallis test, between the different CD4 categories for cervical cancer patients, *p* = 0.767

*p* < 0.05 was considered statistically significant

### Frequency of tregs in participants according to age

**Fig. 5:**
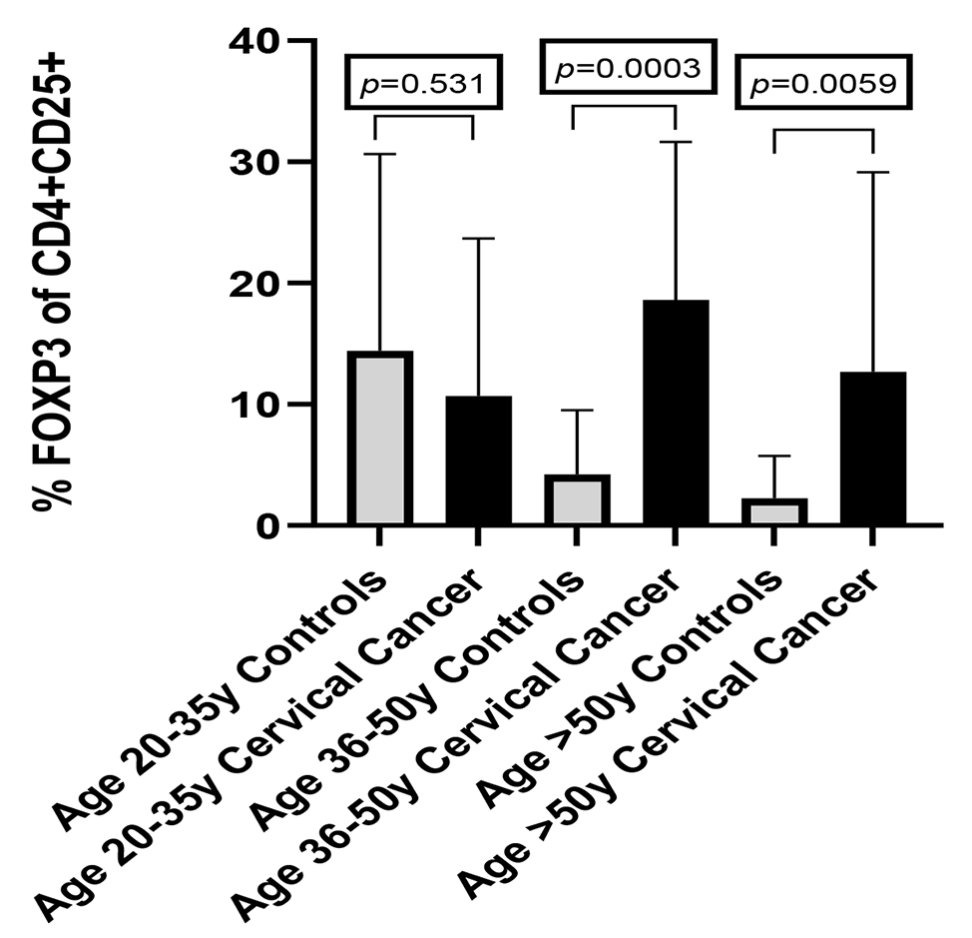
A graphic representation of Treg frequency in cervical cancer (CC) and controls according to age. In the cervical cancer group, Treg frequency was significantly different between the 3 age groups, *p* = 0.0044. (ANOVA) (not shown). In controls, no difference was recorded between any of the age groups (*p* = 0.4136 (not shown). No difference in Treg frequency was found in controls. In comparing Treg frequency between cervical cancer and controls in 36–50 and > 50 age groups, Treg frequency was significantly higher in the cervical cancer women than the controls (*p* = 0.0003 and (*p* = 0.0059), respectively. Bars represent the median, and error bars represent the interquartile range (IQR)

Study participants were divided into three age groups, 20–35 years, 36–50 years and > 50 years, to determine whether age had any influence on the frequency of Tregs (Fig. 5). In women with cervical cancer, a significant difference was observed (*p* = 0.044) (not shown), with the highest levels of Tregs in the 36–50 age group. In controls, no difference was recorded between any of the age groups. Comparing Treg frequency between cervical cancer and controls in the 36–50 years and > 50 year age groups, Treg frequency was significantly higher in women with cervical cancer than controls (*p* = 0.0003 and *p* = 0.0059), respectively (Fig. 5.).

### Logistic regression analysis

Logistic regression analysis was applied to our data to assess the association of cervical cancer with Tregs and other variables (Age, HIV, CD4, HPV genotype, STI, loss of weight and smoking). A strong association of cervical cancer was noted with Tregs, age, HIV, HPV genotypes and loss of weight. STI was also associated with cervical cancer, albeit to a lesser degree. (Table 3).

**Table 3:** Univariate logistic regression analysis of Tregs and relevant variables in cervical cancer and Control participants

Variables	Odd ratio	(CI)	*P* value
Tregs	1.089	(1.034–1.147)	0.001
Age	1.067	(1.041–1.093)	< 0.001
HIV	0.276	(0.154–0.4160	< 0.001
CD4	0.996	(0.994–0.998)	< 0.001
HPV genotype	0.006	(1.602–2.510)	< 0.001
Loss of weight	0.037	(0.17–0.077)	< 0.001
Smoking	1.922	(0.810–4.563)	0.139
STI	0.492	(0.268-0902)	0.022

Multivariate logistic regression analysis was further performed to confirm the true associations. Treg frequency, loss of weight and HPV genotype were revealed as having true association with cervical cancer when controlling for other factors (Table 4).

**Table 4:** Multivariate logistic regression analysis in cervical cancer and Controls participants

Variables	Odd ratio	(CI)	*P* value
Tregs	1.100	(1.018–1.189)	0.016
Age	1.053	(0.972–1.140)	0.206
smoking	7.135	(0.292–174.449)	0.228
STI	0.147	(0.019–1.152)	0.068
Loss of weight	0.018	(0.003–0.124)	< 0.001
HIV	1.670	(0.181–15.426)	0.651
HPV Genotype	2.900	(1.317–6.387)	0.008

## Discussion

Both HPV and HIV are acquired mainly through sexual contact and can result in chronic and latent conditions, persisting for long periods of time in the host by evading the host’s immune system. Nonetheless, the steady and slow reduction of CD4 + T cells in HIV infections correlates with an increased prevalence of HPV infections and HPV-associated cancers [8, 33, 34]. Despite a paucity of information on the role of HIV and HPV infections in cervical cancer patients in eThekwini, KZN reports from earlier studies have revealed a high prevalence of HR HPV and HIV infections in cervical cancer [35, 36] and also from other parts of SA [37–39]. Our study confirms a high prevalence of HR-HPV (98%) and HIV (37%) infections in cervical cancer participants in eThekwini, SA.

We report a significant increase in the frequency of CD4 + CD25 + FoxP3 + T cells (Tregs) in the peripheral blood of cervical cancer participants. These findings are in agreement with other reports [5, 25, 40]. Of note, the Treg amplification contributes to immune suppression propagating cervical cancer progression [28]. Conflicting reports have shown a decrease in Treg frequency in immunodeficient conditions suggesting that Treg production could be impaired [41].

Moreover, HR-HPV infections do not directly lead to cervical cancer development since, in most cases, women are able to resolve the infection spontaneously for about 48 months. However, in a small percentage of women, a delay in the resolution of the infection can lead to the persistence of HPV infections and increase the risk of cervical cancer development [42]. This may be attributed to the immunosuppressive microenvironment emanating from the initial interactions between the host and virus, which subsequently stimulates the induction, development and expansion of HPV-specific Treg dissemination [5].

We also report no statistically significant difference in Treg frequency between stages II and III of cervical cancer (Table 1). This lack of difference may be attributed to either our small sample size or to the maximum induction of Tregs at stage II of the disease development. Nevertheless, it must be noted that our study did not include cervical cancer participants with earlier stages of the disease or with precancerous lesions. Importantly, previous studies have reported increased Tregs in different stages of cervical cancer [25, 28, 43, 44]. Furthermore, Tregs are associated with a lack of spontaneous regression during the early stages of cervical intraepithelial neoplasia [45].

In controls, there was no difference in Treg frequency across the different HPV types (*p* = 0.7788). Notably, in cervical cancer of the HR genotypes, Treg frequency is higher (16.04% ±2.20) compared to controls (3.16% ±0.91), (*p* < 0.0001), Fig. 3. It is, therefore, plausible to hypothesise that Treg anergy may be the contributory factor that is responsible for the host/parasite co-evolution during persistent infections (chronic activation) or high antigen load [33]. This may explain why some individuals cannot eliminate HPV, HIV virions or other pathogen and cancer-infected cells soon after infection. This lack of eliminating viral infections and cancer cells results in the activation of mechanisms that dysregulate Tregs-mediated equilibrium with HPV/HIV creating an imbalance to prolong self-survival and to avoid autoimmune pathologies [33, 46, 47].

Although the role of HR HPV in the progression of cervical cancer is well established, it is not known how the oncogenic HR HPV types contribute to the up-regulation of Treg cells.

Furthermore, the role of HPV type and its influence on Tregs is not well defined. Since our study examined the status of individuals at only one-time point, we are unable to establish the impact or the effect that the different HPV types and Tregs have on the rate of progression of cervical cancer. A longitudinal study will assist in providing a better assessment of the relationship, if any, of HPV types in cervical cancer and Treg proportion. Nevertheless, the immunosuppressive effect arising from the increased levels of Tregs may explain why infections such as HPV in cervical cancer participants cannot easily be resolved. These findings are consistent with previous reports [5, 6, 27, 48].

Variability in Treg frequency in HIV-infected individuals may be attributed to several factors, including HIV factors (HIV types or clades A, B, C, D) or host genetic factors (i.e. race, HLA) [49]. Most reports where Tregs were reduced or did not change with HIV infection were from regions where HIV clade B was the most prevalent [50–52]. In SA, elevated Treg frequency was recorded in a population with a high prevalence of HIV clade C [53]. In a comprehensive, well-designed longitudinal study, Schulze et al. presented their data on the frequency of Tregs across the stages of HIV disease and evaluated the influence of highly active antiretroviral therapy (HAART) [54]. They found a significantly higher frequency of Tregs in HIV-infected individuals compared to the controls, which correlated positively with viral load and negatively with CD4 counts. In addition, they found a compelling association with regard to the quantity and quality of Tregs across different stages of HIV disease. Patients on HAART expressed higher Treg frequency than the non-progressor but lower than the progressor [54]. More recently, Yero et al. have explored the dynamics of Treg frequency in acute HIV infection following antiretroviral treatment [55]. Others have shown mucosal and not peripheral Treg frequency are increased in HIV infection, which is normalised after suppressive HAART [56].

In our study, using univariate regression analysis, we found HIV infection to be associated with cervical cancer (Table 3). However, on further multivariate regression analysis including HPV genotypes, the HIV association with cervical cancer was no longer present, while Treg frequency remained significantly associated with cervical cancer (Table 4). We, therefore, concluded from our study that HIV is not a unique association with cervical cancer but rather HIV infection often correlates with increased Treg frequency, which in turn is strongly associated with cervical cancer.

Recently, low frequency of Tregs was reported in chronically infected HIV-1 patients, suggesting low levels of Tregs occur during immune exhaustion, and can be reversed with antiviral therapy (ART), as proposed [50, 52, 57, 58]. In some cases, higher Treg frequency has been recorded in condylomata acuminate lesions and other diseases [54, 59, 60], whilst in other instances, no change in Treg levels occurs [26, 51]. Discrepancies in results obtained from different studies may be attributed to various flow cytometric gating strategies employed by other researchers.

Since age is linked with a gradual waning of the immune system [61], we tested for Treg frequency in the different age categories. We found a significant increase in Tregs in our cervical cancer participants in the 36–50 age group compared to controls (*p* = 0.044), Fig. 5).

Age did not appear to influence the Treg frequency in the control group (*p* = 0.4136), (Fig. 5). In contrast, others have reported a significant increase in Tregs with age [62, 63]. Santner-Nanan et al. further observed that the age-related increase was essentially from Tregs with an effector memory phenotype, whereas those with a central memory phenotype remained unchanged [62]. This increased effector Treg memory phenotype may explain the immunosuppressive status of aged individuals in our cervical cancer participants. It has been suggested that the accumulation of Treg frequency in older individuals can promote chronic infectious disease reactivation [64].

The strengths of this study are that we provide data on Treg frequency in cervical cancer with HPV and HIV infections. In addition, our study had a large sample size, with both HIV and non-infected HIV cervical cancer. However, the limited number of HIV-infected participants in the control group reduced the strength of our conclusions. Furthermore, a significant limitation was that this study was cross-sectional, and therefore, it was not possible to evaluate the impact of Tregs on the progression of the disease. We could not address the role of HAART treatment since the participants in our study were newly diagnosed with HIV infection. Another limitation was that the kit used for Flow cytometry did not include a marker for live cell/dead cell discrimination such as propidium iodide. The kit contained rather a non-specific/isotype antibody control tube that enabled the removal of the percentage of non-specific binding/dead cells from the FoxP3 binding cell frequency to obtain the live Treg frequency.

Nevertheless, our study provides novel data on peripheral Tregs frequency in pre-HAART treatment. Another limitation of our study is that we used the only marker (Foxp3+) available at that time to study the Treg frequency. Furthermore, we evaluated the frequency of Tregs in peripheral blood and not in tissues. Rios et al. recently reported that since there is a reduction of Treg expressing CD25, using this molecule as a marker for the characterisation of Tregs in HIV patients, especially in tissues, will not be appropriate [53].

Recent experimental data have suggested Tregs derived from CD4 + T cells or Treg subsets can be eliminated in the tumour environment by immunotherapy that is aimed at blocking Foxp3, together with the selective inhibition of other minor Treg subsets [65].

Several approaches to neutralise or eliminate Tregs have formed the basis of the latest innovative methods as part of cutting-edge trends in cancer immunotherapy [66, 67]. It has been recommended that HPV-derived lesions, CIN, which are the precursors of cervical cancer, be considered prime targets for such non-invasive therapies involving the depletion of Tregs [44, 68]. However, as a cautionary note, it has been suggested that peptide-based therapeutic HPV vaccines could prove to be unsafe in-established cervical cancers since they could induce E6/E7 specific CD4 + Tregs in the environment of cancer and in the peripheral blood [33, 69]. Recent trials have shown that the removal or decrease of the number of Tregs prior to cancer immunotherapy increased the survival of patients [70, 71]. FDA-approved agents to remove Tregs are being used as adjuvants in current immunotherapies [72].

## Conclusion

Taken together, our data on the frequency of Tregs from peripheral blood in cervical cancer and control participants are in accordance with the literature showing that Treg frequency is elevated in HIV infection and in cervical cancer. However, this study demonstrates a stronger association of Treg frequency with the presence of cervical cancer than HIV infection. We have provided baseline data showing that high Treg frequency is a strong correlate with cervical cancer. We suggest that Treg frequency in peripheral blood be further evaluated as a tool for monitoring progression between early to late cervical cancer stages and prognosis during cervical cancer treatment.

## Data Availability

Data and materials are available upon request from the corresponding author.
